# Upregulation of Pin1 contributes to alleviation of cognitive dysfunction in diabetic mice

**DOI:** 10.1002/brb3.3336

**Published:** 2023-11-21

**Authors:** Dan Qiu, Tong Liu, Wei Qianping

**Affiliations:** ^1^ Department of Gerontology The First Affiliated Hospital of Chongqing Medical University Chongqing China; ^2^ Bishan District Traditional Chinese Medical Hospital Chongqing China

**Keywords:** cognitive dysfunction, Pin1

## Abstract

**Objective:**

This study aimed to explore the molecular mechanism underlying the role of Pin1 in cognitive dysfunction in diabetic mice.

**Methods:**

Using a streptozotocin‐induced diabetic mouse model, an adeno‐associated virus carrying the *Pin1* gene was used to upregulate Pin1 expression in the hippocampus of diabetic mice. Animal behavior tests and molecular biology techniques were further used to explore the role of Pin1 in cognitive dysfunction in diabetic mice.

**Results:**

Our study demonstrated that upregulation of Pin1 expression increased the phosphorylation of AKT and insulin receptor substrate 1 downstream signaling molecules of the IR‐IGF1R pathway, increased the phosphorylation of GSK‐3β, and concomitantly decreased the phosphorylation of Tau in the hippocampus of diabetic mice, thereby improving the ultrastructural pathology of the hippocampus and further alleviating diabetes‐related cognitive impairment.

**Conclusion:**

Pin1 can improve cognitive dysfunction in diabetic mice.

## Graphical abstract

We observed behavioral changes in mice and used molecular biology and histopathological to demonstrate that Pin1 may play an important role in the onset and development of diabetic encephalopathy by selectively upregulating Pin1 expression.

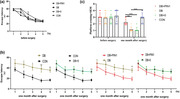

## 1 INTRODUCTION

In 1965, Nielon reported that diabetes is accompanied by changes in brain neurophysiology and structure manifested by mild to moderate cognitive dysfunction and behavioral deficits, known as diabetic encephalopathy (Yang et al., 2014).

In recent years, diabetes‐associated cognitive dysfunction has increased yearly in terms of incidence and become a main focus of diabetes research. The hippocampus is an important region of the central nervous system related to memory. We and others have previously confirmed that the hippocampus is sensitive to persistent high blood sugar levels, which reduce the number of hippocampal neurons and impair cell body morphology and synaptic function, thus leading to cognitive dysfunction (Freude et et al., 2009; L. Ma et al., 2013). Neuronal apoptosis is the main cause of brain dysfunction in diabetes, and dysregulation of the insulin receptor (IR) and insulin‐like growth factor‐1 receptor (IGF‐1R) signaling pathways in the hippocampus plays an important role in the induction of neuronal apoptosis and consequently the occurrence and development of diabetic encephalopathy (Hoffman et al., 2010; Ito et al., 2012).

Peptidyl‐prolyl cis‐trans isomerase NIMA‐interacting 1 (Pin1) is a prolyl isomerase that specifically binds and isomerizes phosphorylated Ser/Thr‐Pro in target proteins, leading to changes in their structure and function, which further drive various cellular processes and participate in the regulation of signaling pathways (Driver et al., 2014; Liou et al., 2003; Pastorino et al., 2006; K. P. Lu et al., 2007). Diabetes is an independent risk factor for Alzheimer's disease (AD) (Ott et al., 1999). One of the common shared mechanisms between diabetic encephalopathy and AD is excessive phosphorylation of tau protein (Guo et al., 2010; Katsumoto et al., 2019; Lanni et al., 2021; Vlassara & Palace, 2003). Recently, a study published in *Nature* reported that downregulation of Pin1 expression can exacerbate the excessive phosphorylation of Tau in an AD mouse model (Liou et al., 2003). Another study conducted abroad showed that Pin1 can catalyze the conversion of the Tau protein structure from a dysfunctional cis structure to a functional trans structure, thereby reducing Tau phosphorylation (Driver et al., 2014; Pastorino et al., 2006). An excessive increase in Tau phosphorylation is an early pathological change in patients with mild cognitive impairment (Lanni et al., 2021; Katsumoto et al., 2019). In addition, a study conducted abroad found that upregulation of Pin1 expression in the liver of obese mice can increase the phosphorylation of the downstream signaling molecules insulin receptor substrate 1 (IRS1) and serine/threonine kinase (AKT), components of the IR/IGF1R pathway, ultimately increasing insulin sensitivity and increasing glucose metabolism (Nakatsu et al., 2011). The phosphorylation of AKT in mouse pheochromocytoma 12 cells induces the phosphorylation of GSK‐3β, resulting in the loss of its activity (Fukumoto et al., 2001; Qian et al., 2021). The level of phosphorylated GSK‐3β is reduced in an AD mouse model, further leading to the excessive phosphorylation of Tau (Angelucci & Hort, 2017; Butterfield et al., 2006; Chen et al., 2015; Grison et al., 2011; Hall et al., 2011; Liao & Hung, 2010; P. J. Lu et al., 1999; S. L. Ma et al., 2012; Pastorino et al., 2006). Excessive phosphorylation of Tau causes the formation of neurofibrillary tangles (NFTs), which is one of the pathological mechanisms underlying diabetic encephalopathy (Guo et al., 2010). Upregulation of Pin1 expression in an AD mouse model can reduce the excessive phosphorylation of Tau (Angelucci & Hort, 2017; Butterfield et al., 2006; Chen et al., 2015; Grison et al., 2011; Hall et al., 2011; Liao & Hung, 2010; P. J. Lu et al., 1999; S. L. Ma et al., 2012; Pastorino et al., 2006). Therefore, it is hypothesized that Pin1 may play an important role in protecting against diabetic encephalopathy. The aim of this study was to explore the critical role of Pin1 in the development of cognitive dysfunction in a diabetic mouse model.

## 2 MATERIALS AND METHODS

### 2.1 Ethical statement

All animals used in the study were handled according to the International Council of Medical Scientific Organizations International Guidelines for Biomedical Research involving Animals. The animal experiment was approved by the Animal Ethics Committee of Chongqing Medical University. The animals were housed in the Experimental Animal Center of Chongqing Medical University, fed a standard laboratory diet, allowed ad libitum access to drinking water in a clean environment, and maintained on a constant 12‐h:12‐h light/dark cycle. At the end of the experiment, the mice were sacrificed after deep anesthesia with an intraperitoneal injection of 3% pentobarbital sodium.

### 2.2 Animal model

Male C57BL/6 mice (3−8 weeks old, 16−22 g) were purchased from Hunan Slack Jingda Experimental Animal Co, Ltd. One month following acclimation, after fasting for 8 h, mice were randomly selected for diabetes modelling by a single intraperitoneal (i.p.) injection of a high dose of streptozotocin (STZ,180 mg/kg) freshly dissolved in 0.1 mmol/L sodium citrate‐hydrochloric acid buffer (pH 4.5) (STZ, 50 mg/mL, Sigma‒Aldrich), while mice injected with an equal volume of STZ‐free buffer solution served as the control group. Three days later, the fasting blood glucose level in tail vein blood samples was measured using a blood glucose meter (Roche Diagnosis). Mice with blood glucose levels greater than 16.7 mmol/L were considered diabetic mice (Guo et al., 2010). Blood glucose levels and body weight were measured once a week. The mice in the experimental group were further randomly divided into the diabetic (DB) group, diabetic sham treatment (DB + 0) group, experimental (DB + Pin1) group, and control group. There were 10 mice in each group.

### 2.3 Stereotactic surgery

At 11 weeks after STZ injection, animals were anaesthetized by intraperitoneal injection of 4% chloral hydrate. Adeno‐associated virus (AAV) vectors carrying the *Pin1* gene (NCBI ID NM_023371) or GFP alone were obtained from GeneChem and microinjected into the bilateral hippocampus. The stereotaxic coordinates were determined from a mouse brain map: AP: −2.0 mm, ML: ±1.2 mm, and DV: −2.0 mm (Paxinos & Franklin, 2001). Mice in the diabetic sham treatment (DB + 0) group were injected with the same dose of empty AAV vectors. All mice were injected with 1.0 μL of virus into the hippocampus of each hemisphere over 4 min (0.25 μL/min). After each injection, the syringe was kept in place for 1 min and then slowly removed.

### 2.4 Water maze test

The Morris water maze test was performed based on the studies by A. M. Barron and Rachna Gupm (Barron et al., 2010; Gupta et al., 2012; Vorhees & Williams, 2006). The water maze consisted of a circular pool with a diameter of 120 cm. There was a black curtain around the maze to darken the area. The wall of the pool was black, and four entry points were marked with signs on the wall. According to the locations of these four signs, the pool was divided into four quadrants. A movable circular platform with a diameter of 10 cm in the pool was placed in one of the quadrants. The position remained unchanged during the spatial learning phase and changed during the exploration phase. The water level was kept below the height of the platform by approximately 1 cm. Milk powder was evenly dissolved in water to ensure that the water was white and opaque, and the water temperature was maintained at +23°C (Gupta et al., 2012).

During the 5‐day spatial learning phase, the mice were placed in the water facing the wall at one of our entry points and were given 60 s to find the hidden platforms. The latency of each mouse to find the platform (escape latency) was recorded. If mice found the platform autonomously, they were allowed to stay on the platform for 10 s. If a mouse did not find the platform within 60 s, the experiment ended, the escape latency was recorded as 60 s, and the mouse was guided to the platform, where it was kept for 10 s before being returned to its cage.

During the exploration phase, the platform was removed. The mice were placed in the water as described above, and the number of times the mice entered the place where the platform was placed within 60 s (the number of platform crossings) and the time spent in the target quadrant (Vorhees & Williams, 2006) were recorded.

### 2.5 Hematoxylin‐eosin and immunofluorescence staining

#### 2.5.1 Hematoxylin–eosin staining

At 7 weeks after virus injection, the mice were deeply anaesthetized with 4% chloral hydrate, and the brains were removed after cardiac perfusion with phosphate‐buffered saline (PBS) and 4% paraformaldehyde. The brain was immersed in 4% paraformaldehyde at 4°C for 24 h and embedded in paraffin. Five‐micrometer paraffin‐embedded sections were prepared for hematoxylin–eosin staining.

#### 2.5.2 Immunofluorescence staining

After dewaxing, hydration, and antigen retrieval, paraffin sections of hippocampal tissues were blocked with 0.1% BSA (bovine serum albumin) in PBS, followed by incubation with primary antibody (rabbit anti‐Pin1 antibody, 1:200, Proteintech, 10495‐1‐AP) overnight at 4°C. Following washing three times with PBS, the sections were incubated with biotin‐labeled goat anti‐rabbit secondary antibody at room temperature for 60 min, and the nuclei were stained with DAPI (4',6‐diamidino‐2‐phenylindole). Images were captured under a fluorescence microscope.

### 2.6 Real‐time qRT‐PCR

Hippocampal tissues were fully ground in liquid nitrogen, and 20 mg of tissue was utilized for total RNA extraction. The concentration and purity of the RNA samples were determined by an ultramicrospectrophotometer (NanoDrop 2000), and RNA with an A260/280 peak between 1.8 and 2.0 was selected for subsequent experiments. The RNA was reverse transcribed into cDNA using a reverse transcription kit. Finally, a PCR plate containing 2 × SYBR Green qPCR Master Mix, primers, cDNA, and enzyme‐free water was used for PCR amplification. The primers for qPCR were designed and synthesized by Xavier Company. The sequences of primers for Pin1 were as follows: upstream primer: 5′‐CTGTCGCCTCCTGTTCCTACT‐3′; downstream primer: 5′‐ATTCTTGCTGCTGCCTCCAA‐3′. GAPDH was selected as the internal reference, and the sequences of the GAPDH primers were as follows: upstream primer: 5′‐CCTCGTCCCGTAGACAAAATG‐3′; downstream primer: 5′‐TGAGGTCAATGAAGGGGTCGT‐3′.

### 2.7 Western blot analysis

Western blotting was performed as described previously (Colley et al., 2009). The membranes were reprobed with an antibody specific for GAPDH (Saiweier) as an internal control. Semiquantitative analysis of the bands was performed using Quantity One software version 4.6.2 (Bio‐Rad). ImageJ software (Vision: v.1.52p) was used to analyze the target band and the corresponding internal reference band and perform semiquantitative gray analysis of the two bands. The specific primary antibodies included a rabbit polyclonal antibody against Pin1 (1:1000, Proteintech, 10495‐1‐AP), a rabbit polyclonal antibody against phospho‐tau (Thr231 1:800, AF3147), a rabbit polyclonal antibody against phospho‐GSK‐3β (Ser9 1:800, AF2016), a rabbit polyclonal antibody against phospho‐AKT (Ser473 1:800 ImmunoWay Biotechnology, Inc, YT0176), a rabbit polyclonal antibody against phospho‐IRS1 (Ser307 1:800 ImmunoWay Biotechnology, Inc), and a rabbit polyclonal antibody against GAPDH (1:2000, AF7021).

### 2.8 Transmission electron microscopy

Hippocampal tissue was taken after perfusion with PBS and 2.5% glutaraldehyde solution (25% glutaraldehyde solution, 0.2 M phosphate buffer, 3 mM MgCl_2_) for fixation. For observation of possible changes in synapses and other ultrastructural changes, sections containing either the upper third or the middle third of the hippocampal CA1 radiation layer were observed by electron microscopy (Zhou et al., 2010). The synapse density was determined by software Adobe Photoshop (Version: 20.0.2 20181219.r.30).

### 2.9 Statistical analysis

All results were analyzed using SPSS 26.0 (SPSS, Inc.) and are presented as the mean ± SEM. One‐way analysis of variance (ANOVA) was conducted. If a significant difference was found, Tukey's HSD (equal variance) or Dunnett's T3 (unequal variance) test was conducted to determine which groups differed significantly. All statistical tests were two‐sided with significance set at *p* < .05.

## 3 RESULTS

Since surgical trauma can also affect animal behavior, we performed the classic Morris water maze (MWM) test to evaluate the spatial and positional abilities of the mice. The results showed that surgery had no significant effect on the spatial or positional learning and memory abilities of the mice.

### 3.1 General characteristics of mice in each group

**FIGURE 1:**
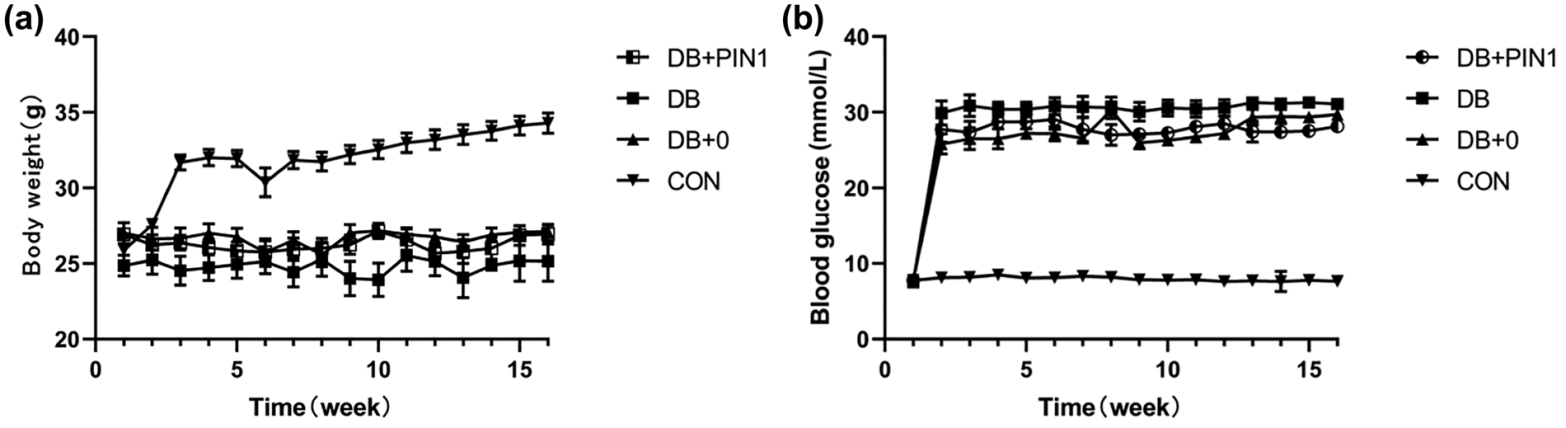
The body weight (a) and blood glucose levels (b) of C57BL/6 mice were measured once a week. Blood samples were collected from the tails of mice under fasting conditions. Body weight was measured in grams, and blood glucose levels were measured using a blood glucose meter. The data are expressed as the mean ± SEM (*n* = 10). CON, normal control group; DB, diabetic group; DB + 0, sham treatment group; DB + Pin1, experimental group.

The mice in different groups had similar body weights and blood glucose levels before STZ treatment. One week after STZ injection, the blood glucose levels of all diabetic mice (DB group, DB + 0 group, and DB + Pin1 group) were greater than 16.7 mmol/L (Figure 1b), suggesting the successful establishment of the diabetic mouse model. Moreover, the body weight of the diabetic mice was lower than that of the control mice (Figure 1a). Throughout the whole experiment, diabetic mice exhibited persistent hyperglycemia (Figure 1b) and typical symptoms of diabetes, such as polydipsia, polyphagia, polyuria, weight loss, fatigue, irritability, and apathy.

### 3.2 Effects of high blood glucose levels and upregulation of Pin1 gene expression on cognitive function

**FIGURE 2:**
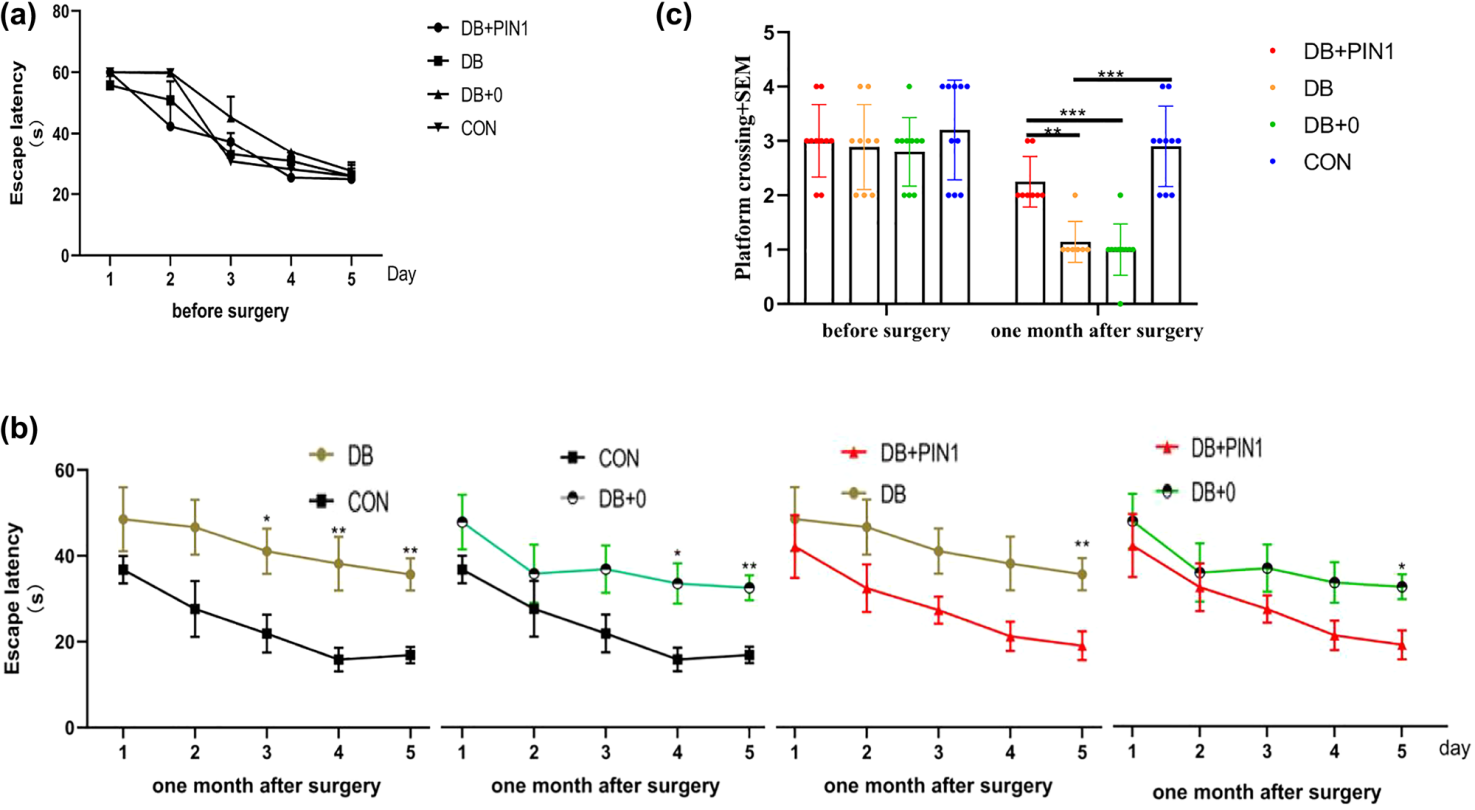
The Morris water maze (MWM) test was performed before intrahippocampal injection surgery and 1 month after surgery in adult male mice. Learning acquisition curve of adult male mice from 1 month after surgery showing the effects of streptozotocin (STZ) and the protective effects of Pin1 on the mean escape time to reach the hidden platform (escape latency) over consecutive trials in the MWM test. In the probe trials, the number of platform crossings (c) was recorded. The data are expressed as the mean ± SEM (*n* = 7–10 per group). Statistical analysis was performed using one‐way analysis of variance followed by Tukey's HSD test: (b, c) (*p < .05; **p < .01; ***p < .001). CON, normal control group; DB, diabetic group; DB + 0, sham treatment group; DB + Pin1, experimental group.

There was no significant difference in the average escape latency between the DB group and the DB + 0 group after surgery (Figure 2b), indicating that stereotactic surgery and AAV injection had little effect on performance in the MWM test. ANOVA revealed that 1 month after the operation, the average escape latencies on the fourth and the fifth days were significantly different among the groups (the fourth day: *F*(3,31) = 5.806, *p* < .01; the fifth day: *F*(3.31) = 10.258, *p* < .001) with the BD and DB + 0 groups exhibiting significantly longer escape latencies than the control group (the fourth day: *p* < .01 and *p* < .05;the fifth day: all, *p* < .01, Figure 2b). ANOVA also revealed that there were significant differences in the number of platform crossings among the groups (*F*(3, 31) = 25.706, *p* < .001), with the DB group making significantly fewer platform crossings than the control group (*p* < .001, Figure 4c), indicating that the spatial memory function of the diabetic mice was impaired and that spatial memory impairment can be used as an indicator of diabetic encephalopathy. According to ANOVA, the average escape latency on the fifth day significantly differed among the groups (*F*(3, 31) = 10.258, *p* < .001) with the DB + Pin1 group showing a significantly shorter escape latency than the DB and DB + 0 groups (*p* < .01 and *p* < .05, respectively, Figure 2b). Furthermore, ANOVA revealed that the number of platform crossings significantly differed among the groups (*F*(3, 31) = 25.706, *p* < .001), with the BD + Pin1 group crossing the platform location significantly more times than the DB and DB + 0 groups (*p* < .01 and *p* < .001, respectively, Figure 2c), suggesting that overexpression of Pin1 can alleviate diabetic encephalopathy.

### 3.3 The effect of Pin1 upregulation on IGF1R‐IR signal transduction

**FIGURE 3:**
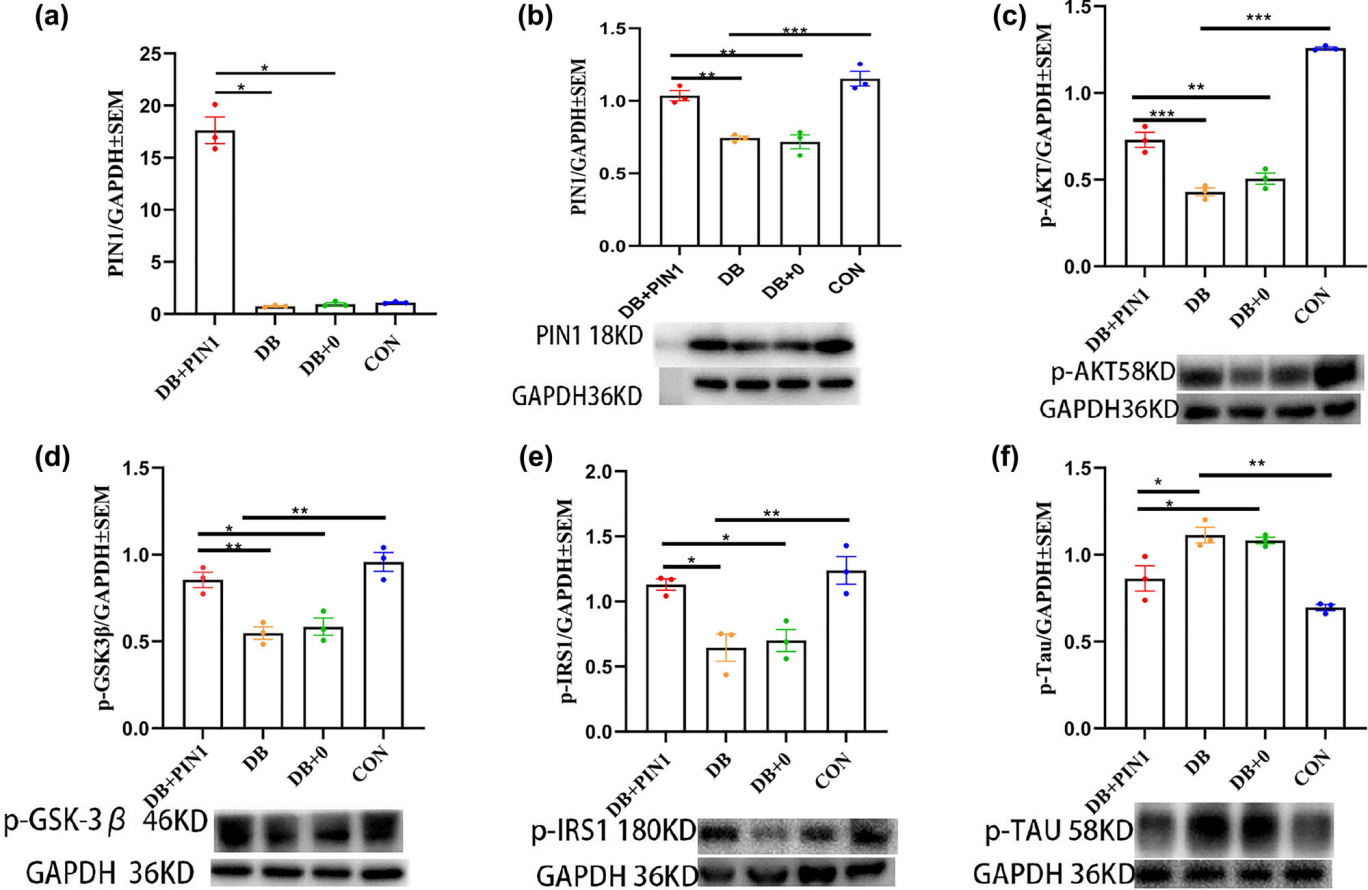
Pin1 mRNA (a) was measured by real‐time qRT‐PCR, and the protein levels of Pin1 (b), p‐AKT (C), p‐GSK‐3β (d), p‐IRS1 (e), and p‐Tau (f) were measured by western blotting. The results of three independent experiments were quantified, and the histogram shows the average mRNA or protein expression in each group. The data are expressed as the mean ± SEM (*n* = 3). Statistical analysis was performed using one‐way analysis of variance followed by Tukey's HSD test (c–f) or Dunnett's T3 test (A and B) (*p < .05; **p < .01; ***p < .001). CON, normal control group; DB, diabetic group; DB + 0, sham treatment group; DB + Pin1, experimental group.

As expected, according to ANOVA, Pin1 mRNA and protein levels in the hippocampus of mice were significantly different among the groups (mRNA: *F*(3,8) = 169.444, *p* < .001; protein: *F*(3, 8) = 29.090, *p* < .001), and the DB + Pin1 group exhibited significantly higher Pin1 mRNA and protein levels than the DB and DB + 0 groups (all *p* < 05, Figure 3a; all *p* < .01, Figure 3b). These results demonstrate that local microinjection of Pin1‐expressing AAV vectors can specifically upregulate Pin1 gene expression at the mRNA and protein levels. To further determine the role of Pin1 in regulating the IGF1R‐mediated signal transduction pathway, we measured the levels of phosphorylated forms of insulin receptor substrate 1 (p‐IRS1, Ser307), serine/threonine kinase Akt (p‐Akt, Ser473), Tau (p‐Tau, Thr231), and glycogen synthase kinase 3β (p‐GSK‐3β, Ser9) in the hippocampus by western blotting. According to ANOVA, the levels of p‐Akt, p‐GSK‐3β, and p‐IRS1 were significantly different among the groups (p‐Akt:*F*(3, 8) = 159.887, *p* < .001; p‐GSK‐3β: *F*(3, 8) = 18.898, *p* < .01; p‐IRS1: *F*(3, 8) = 11.557, *p* < .01), with p‐Akt, p‐GSK‐3β, and p‐IRS1 levels being significantly higher in the DB + Pin1 group than in the DB and DB + 0 groups (all *p* < .05, respectively, Figure 3c–e) and significantly higher in the control group than in the DB group (all *p* < .05, Figure 3c–e). ANOVA also revealed that the level of p‐Tau significantly differed among the groups (*F*(3.8) = 19.054, *p* < .01), with the DB + Pin1 group exhibiting significantly lower levels of p‐Tau than the DB and DB + 0 groups (all *p* < .05, Figure 2f) and the control group showing significantly lower levels of p‐Tau than the DB group (*p* < .01, Figure 2f). These results suggest that upregulation of Pin1 expression promotes the activation of the IR‐IGF1R‐IRS1‐PI3K/Akt signaling pathway, resulting in an increase in inactive phosphorylated GSK‐3β levels and a concomitant decrease in p‐Tau protein levels in the hippocampus of DB mice.

### 3.4 Hematoxylin‐eosin staining, immunofluorescence staining, and electron microscopy of the hippocampus

**FIGURE 4:**
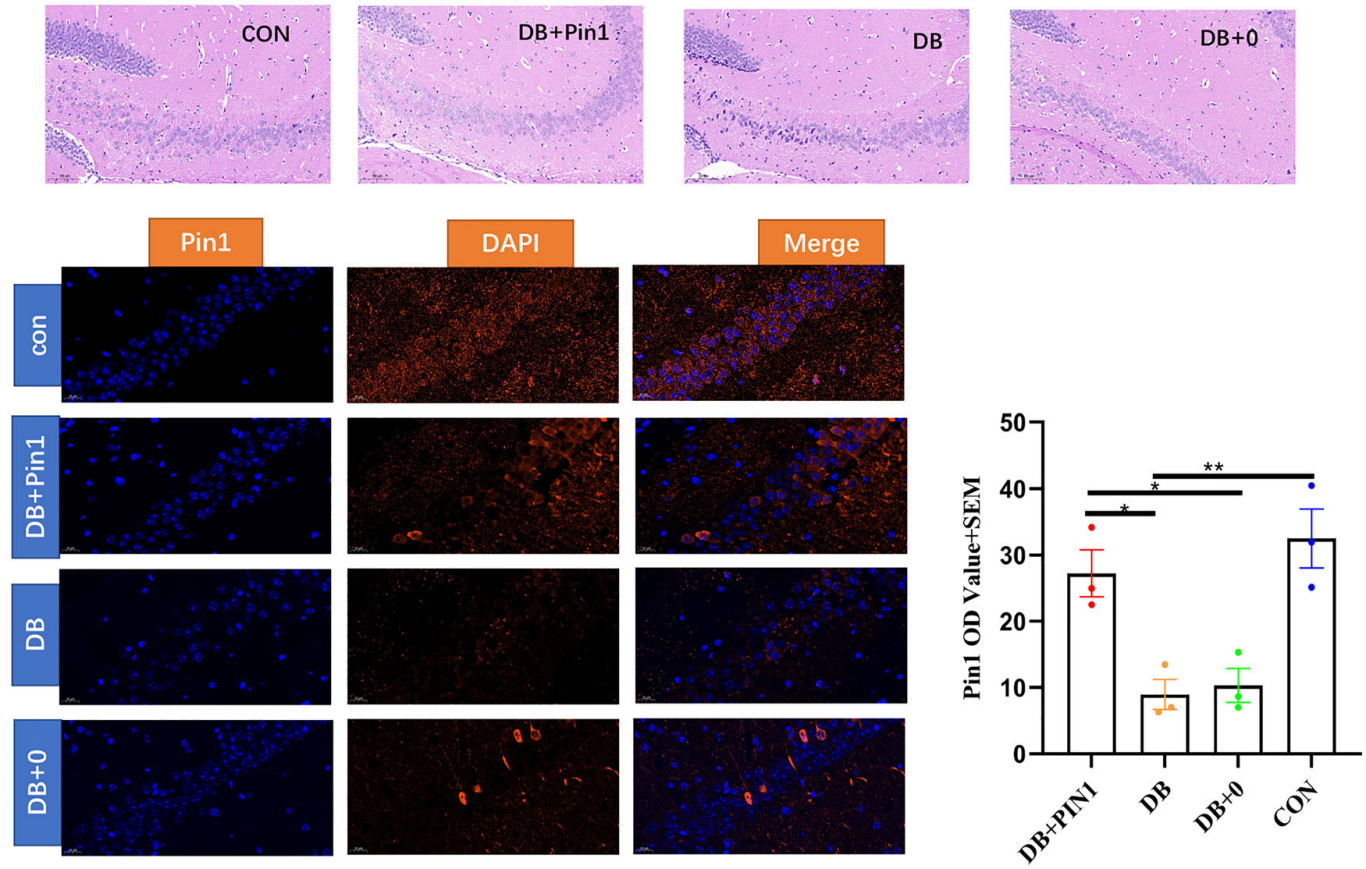
Compared with those in the DB group, DB + Pin1 group and CON group, hippocampal neurons in the DB group showed increased senescence, a decrease in number, and a disordered arrangement. The neurons in the DB + Pin1 group were more neatly arranged than those in the DB and DB + 0 groups .The mean Pin1 OD was determined by ImageJ (*V1.8.0.112*), and the data are expressed as the mean ± SEM (*n* = 3). Statistical analysis was performed using one‐way analysis of variance followed by Tukey's HSD test (5) (*p < .05; **p < .01; ***p < .001). CON, normal control group; DB, diabetic group; DB + 0, sham treatment group; DB + Pin1, experimental group.

**FIGURE 5:**
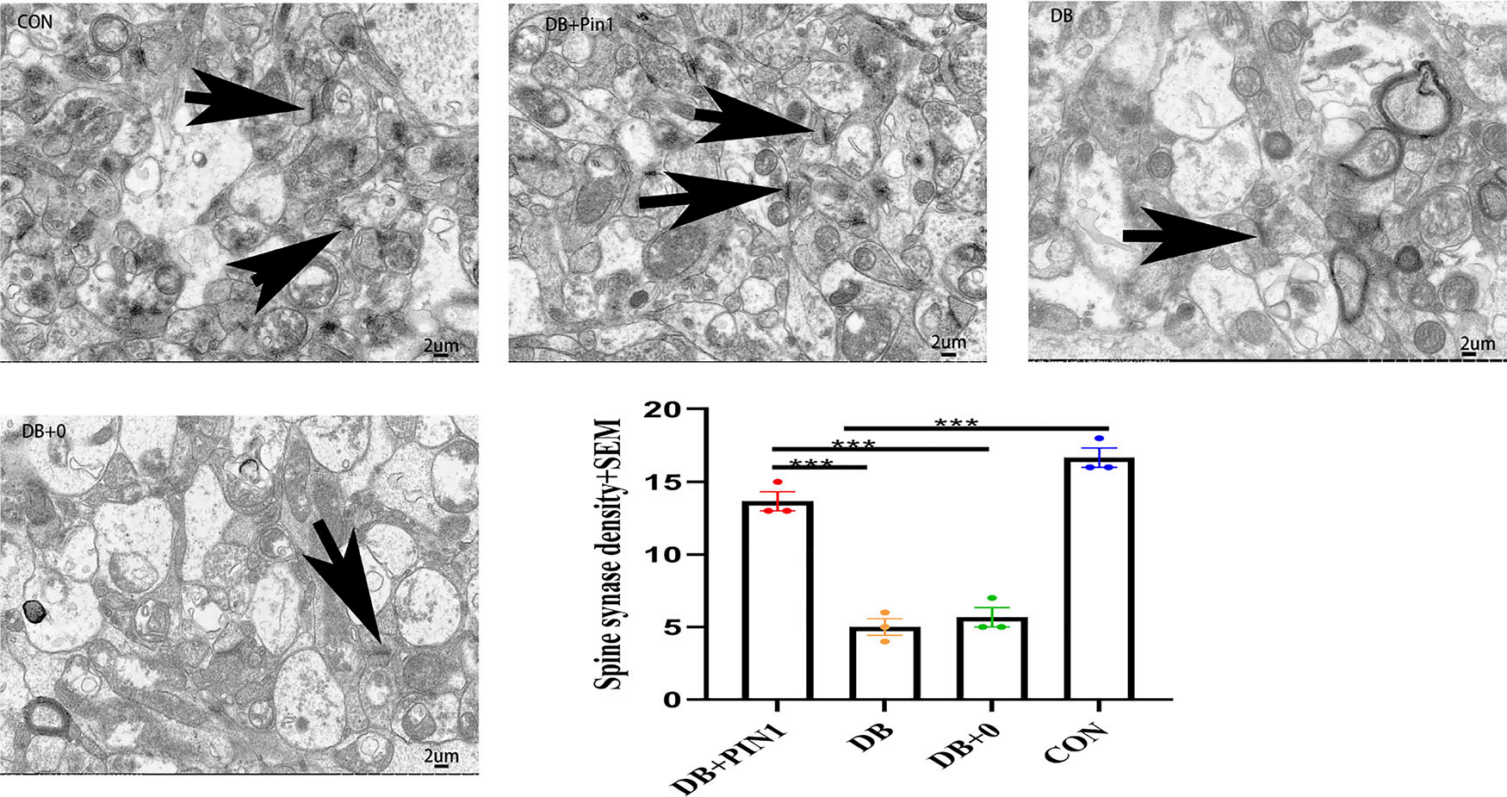
Specimens of the CA1 region of the mouse hippocampus were prepared for electron microscopy specimens. The arrow points to a morphologically intact synapse. In the diabetic group, hyperglycemia resulted in significant decreases in synaptic density and axonal swelling after the injection of streptozotocin. The spine density was determined by magnifying the image 8000 times, and the data are expressed as the mean ± SEM (*n* = 3). Statistical analysis was performed using one‐way analysis of variance followed by Tukey's HSD test (4) (*p < .05; **p < .01; ***p < .001). CON, normal control group; DB, diabetic group; DB + 0, sham treatment group; DB + Pin1, experimental group.

Previous studies have shown that persistent hyperglycemia can reduce the number of neurons exacerbate neuronal damage and that cells disrupt disordered cell arrangement in the hippocampal pyramidal cell layer. However, no significant pathological changes were observed in terms of cell morphology or nerve cell damage in the hippocampus of mice from the DB + Pin1 group (Figure 4). Immunofluorescence staining showed that Pin1 was localized in the cytoplasm and membrane of neural cells. Semiquantitative analysis of the immunofluorescence results showed that the mean Pin1 OD in the hippocampus significantly differed among the groups (ANOVA: *F*(3, 12) = 12.880, *p* < .01), with the DB + Pin1 group exhibiting a significantly higher mean Pin1 OD than the DB and DB + 0 groups (all *p* < .05, Figure 4), and the control group showing a significantly higher mean Pin1 OD than the DB groups (*p* < .01, Figure 4). The electron microscopy results showed that the number of synapses in the hippocampal CA1 region significantly differed among the groups (ANOVA; *F*(3, 8) = 81.133, *p* < .001), with the number of synapses being significantly higher in the DB + Pin1 group than in the DB and DB + 0 groups (all *p* < .001, Figure 5) and significantly higher in the control group than in the DB group (*p* < .001, Figure 5). These results further imply that increased Pin1 expression in the hippocampus can protect against DB‐associated pathological changes in the central nervous system.

## 4 DISCUSSION

### 4.1 Upregulation of Pin1 expression can alleviate diabetic cognitive dysfunction

Pin1 is a protein phosphatase that specifically binds phosphorylated Ser/Thr‐Pro residues (Driver et al., 2014; Liou et al., 2003; K. P. Lu et al., 2007; Pastorino et al., 2006), and it is related to the pathogenesis of a variety of human neurodegenerative diseases. Pin1 is downregulated in the hippocampus and cortex of temporal lobe epilepsy (TLE) mouse models, which leads to an increase in N‐methyl‐d‐aspartate receptor complex formation, contributing to neuronal injury and apoptosis (Tang et al., 2017). Previous studies have shown that Tau plays an important role in microtubule stabilization, axon development, membrane interactions, and axonal transport. Under pathological conditions, excessive phosphorylation of Tau inhibits its binding with microtubules, leading to microtubule instability and ultimately resulting in neuronal apoptosis (Gong & Iqbal, 2008; Metin‐Armağan et al., 2018). In the brains of AD) patients, the phosphorylation level of Tau is three to four times higher than that in the brains of normal adults (Gong & Iqbal, 2008). Studies have found that the downregulation of hippocampal Pin1 expression in an AD mouse model leads to a reduction in GSK‐3β phosphorylation, resulting in an increase in Tau phosphorylation. Another study showed that downregulation of Pin1 expression can directly increase the excessive phosphorylation of Tau, exacerbating neuronal toxicity and apoptosis (Driver et al., 2014; Hegde et al., 2019; Pastorino et al., 2006). Hegde et al. (2019) demonstrated that dysfunction of the IR/IGF‐1R pathway in an AD mouse model can cause a decrease in GSK‐3β phosphorylation, leading to an increase in Tau protein phosphorylation and exacerbating neuronal toxicity, resulting in neuronal apoptosis. Increasing evidence suggests that increased expression of GSK‐3β is associated with synaptic dysfunction in AD (Facci & Skaper, 2006; Peineau et al., 2007), Tau and β‐amyloid protein aggregation (Llorens‐Marítin et al., 2014; Pérez et al., 2002), and neuroinflammation (Michelucci et al., 2009). Previous studies have shown that the increased expression of AKT phosphorylation in pheochromocytoma 12 cells can cause an increase in GSK‐3β phosphorylation levels (Fukumoto et al., 2001; Qian et al., 2021). Another study has shown that high‐fat diet mice have significantly increased expression of Pin1 in the liver, which can increase the phosphorylation levels of the downstream IR/IGF1R signaling pathway proteins IRS1 and AKT (Nakatsu et al., 2011).

**FIGURE 6:**
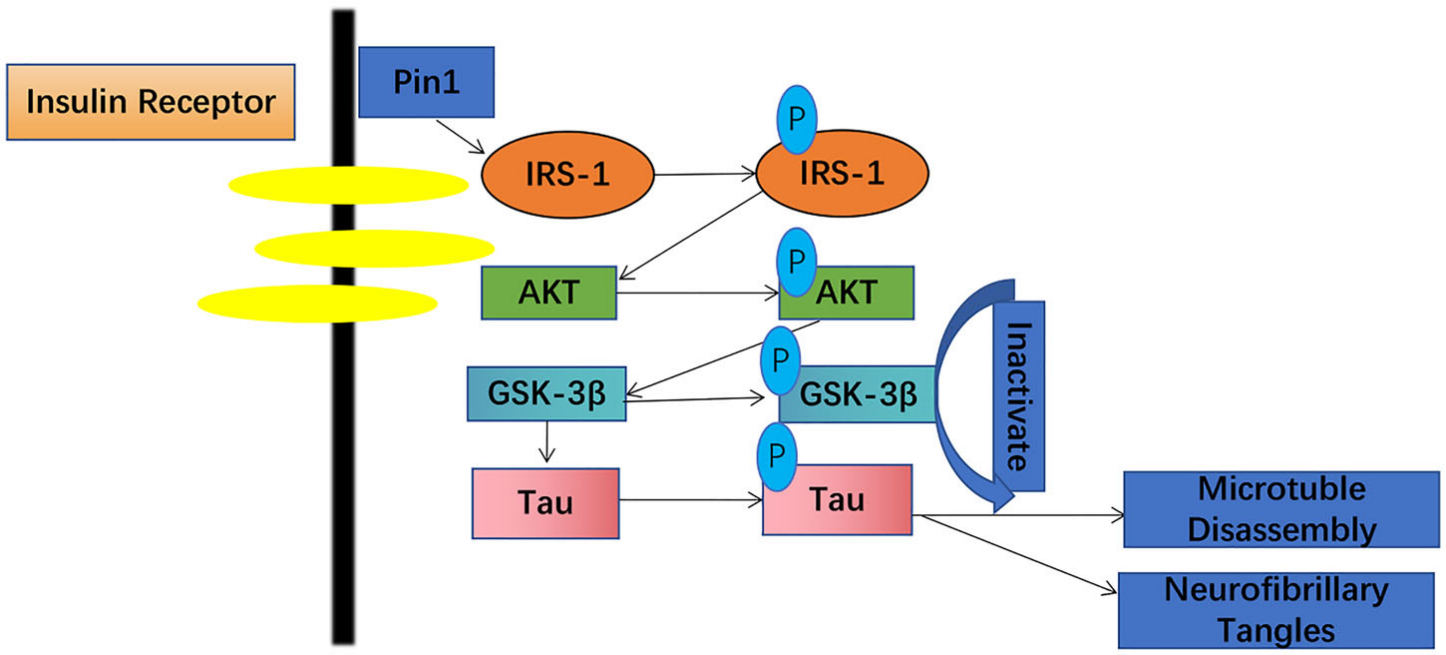
In our study, targeted enhancement of Pin1 expression in the hippocampus of diabetic mice led to upregulation of p‐IRS‐1, p‐AKT, and p‐GSK‐3β expression, downregulation of p‐Tau expression, inactivation of p‐GSK‐3β, and subsequently reduced hyperphosphorylation of Tau, resulting in decreased neurotoxicity.

Our study has shown that Pin1 expression is downregulated in the hippocampus of mice with diabetic encephalopathy, leading to a decrease in the phosphorylation of IRS1, AKT, and GSK‐3β, which are downstream of the IR/IGF1R pathway, an increase in Tau phosphorylation and exacerbation of neuronal apoptosis, resulting in cognitive dysfunction. Upregulating Pin1 expression in the hippocampus of mice with diabetic encephalopathy by stereotaxic injection of an AAV vector significantly increased the phosphorylation of IRS1, Akt, and GSK‐3β, which are downstream of the IR/IGF1R signaling pathway, while significantly decreasing Tau phosphorylation (Figure 6). In addition, upregulating Pin1 expression could directly inhibit the excessive phosphorylation of Tau, ultimately reducing hippocampal neuronal apoptosis and alleviating cognitive dysfunction in mice with diabetic encephalopathy. Therefore, Pin1 may play an important role in the occurrence and development of cognitive dysfunction in diabetes.

## AUTHOR CONTRIBUTIONS

**Dan Qiu**: Data curation; formal analysis; investigation; methodology; visualization; writing—original draft. **Tong Liu**: Methodology. **Qianping Wei**: Conceptualization; funding acquisition; methodology; supervision; writing—review and editing.

## CONFLICT OF INTEREST STATEMENT

The authors declare no conflicts of interest.

### PEER REVIEW

The peer review history for this article is available at https://publons.com/publon/10.1002/brb3.3336.

## Data Availability

The datasets generated and analyzed during the current study are available in the repository (https://orcid.org/0009‐0004‐7477‐5391). Dan Qiu should be contacted if someone wants to request the data from this study (961802630@qq.com).
